# Natural killer cell immunotypes related to COVID-19 disease severity

**DOI:** 10.1126/sciimmunol.abd6832

**Published:** 2020-08-21

**Authors:** Christopher Maucourant, Iva Filipovic, Andrea Ponzetta, Soo Aleman, Martin Cornillet, Laura Hertwig, Benedikt Strunz, Antonio Lentini, Björn Reinius, Demi Brownlie, Angelica Cuapio, Eivind Heggernes Ask, Ryan M. Hull, Alvaro Haroun-Izquierdo, Marie Schaffer, Jonas Klingström, Elin Folkesson, Marcus Buggert, Johan K. Sandberg, Lars I. Eriksson, Olav Rooyackers, Hans-Gustaf Ljunggren, Karl-Johan Malmberg, Jakob Michaëlsson, Nicole Marquardt, Quirin Hammer, Kristoffer Strålin, Niklas K. Björkström, The Karolinska COVID-19 Study Group

**Affiliations:** 1 Center for Infectious Medicine, Department of Medicine Huddinge, Karolinska Institutet, Karolinska University Hospital, Stockholm, Sweden.; 2 Department of Infectious Diseases, Karolinska University Hospital, Stockholm, Sweden.; 3 Division of Infectious Diseases and Dermatology, Department of Medicine Huddinge, Karolinska Institutet, Stockholm, Sweden.; 4 Department of Medical Biochemistry and Biophysics, Karolinska Institutet, Stockholm, Sweden.; 5 Department of Cancer Immunology, Institute for Cancer Research, Oslo University Hospital, Oslo, Norway.; 6 K.G. Jebsen Centre for Cancer Immunotherapy, Institute of Clinical Medicine, University of Oslo, Oslo, Norway.; 7 SciLifeLab, Department of Microbiology, Tumor and Cell Biology, Karolinska Institutet, Stockholm, Sweden.; 8 Division of Infectious Diseases, Department of Medicine, Solna, Karolinska Institutet, Stockholm, Sweden.; 9 Department of Physiology and Pharmacology, Section for Anesthesiology and Intensive Care, Karolinska Institutet, Stockholm, Sweden.; 10 Function Perioperative Medicine and Intensive Care, Karolinska University Hospital, Stockholm, Sweden.; 11 Department of Clinical Science, Intervention, and Technology (CLINTEC), Division for Anesthesiology and Intensive Care, Karolinska Institutet, Stockholm, Sweden.

## Abstract

Understanding innate immune responses in coronavirus disease 2019 (COVID-19) is important to decipher mechanisms of host responses and interpret disease pathogenesis. Natural killer (NK) cells are innate effector lymphocytes that respond to acute viral infections but might also contribute to immunopathology. Using 28-color flow cytometry, we here reveal strong NK cell activation across distinct subsets in peripheral blood of COVID-19 patients. This pattern was mirrored in single-cell RNA sequencing signatures of NK cells in bronchoalveolar lavage from COVID-19 patients. Unsupervised high-dimensional analysis of peripheral blood NK cells furthermore identified distinct NK cell immunotypes that were linked to disease severity. Hallmarks of these immunotypes were high expression of perforin, NKG2C, and Ksp37, reflecting increased presence of adaptive NK cells in circulation of patients with severe disease. Last, arming of CD56^bright^ NK cells was observed across COVID-19 disease states, driven by a defined protein-protein interaction network of inflammatory soluble factors. This study provides a detailed map of the NK cell activation landscape in COVID-19 disease.

## Activated NK cells in severe COVID-19

Natural killer (NK) cells are cytotoxic lymphocytes that provide innate immune defense against viral infections and cancer, but little is known about their involvement in the host response to COVID-19. Maucourant *et al.* used high-dimensional flow cytometry to characterize NK cells in patients with moderate or severe COVID-19. SARS-CoV-2 infection was associated with fewer blood NK cells but a higher activation state in circulating NK cells. Severe COVID-19 resulted in an increase in “armed” NK cells containing high levels of cytotoxic proteins such as perforin. The adaptive NK subset was markedly expanded in a subset of severe patients. These findings lay the groundwork for future studies examining the mechanisms of NK cell activation in COVID-19 and their potential roles in host protection and immunopathology.

## Teaser

The NK cell activation landscape in acute SARS-CoV-2 infection is associated with COVID-19 disease severity.

## INTRODUCTION

The ongoing SARS-CoV-2 pandemic is presenting the human population with profound challenges. SARS-CoV-2 can cause coronavirus disease 2019 (COVID-19) disease, which, in the worst cases, leads to severe manifestations such as acute respiratory distress syndrome, multi-organ failure, and death (*1*). These manifestations may be caused by hyperactivated and misdirected immune responses. High levels of interleukin-6 (IL-6) and an ensuing cytokine storm in the absence of appropriate type I and III interferon responses associate with severe COVID-19 disease (*1*–*6*). Whereas emerging reports suggest that protective immunity is formed in convalescent patients with both neutralizing antibodies and SARS-CoV-2–specific T cells (*7*–*9*), considerably less is known about early innate lymphocyte responses toward the SARS-CoV-2 infection and how they relate to host responses and disease progression. In this study, we evaluated natural killer (NK) cell activation in the context of SARS-CoV-2 infection and resulting COVID-19 disease.

NK cells are innate effector lymphocytes that are typically divided into cytokine-producing CD56^bright^ NK cells and cytotoxic CD56^dim^ NK cells (*10*). Evidence for a direct role of NK cells in protection against viral infections comes from patients with selective NK cell deficiencies, as these develop fulminant viral infections, predominantly with herpes viruses (*11*, *12*). Human NK cells have also been shown to rapidly respond during the acute phase of infections in humans with hantavirus, tick-borne encephalitis virus, influenza A virus (IAV), and dengue virus, as well as after vaccination with live-attenuated yellow fever (*13*–*16*). NK cells not only have the capacity to directly target and kill infected cells but also can influence adaptive T cell responses. The degree of NK cell activation can function as a rheostat in regulating T cells, where a certain level of NK cell activation might promote infection control, while another degree of activation is related to immunopathology (*17*). Compared with peripheral blood, the human lung is enriched in NK cells (*16*, *18*, *19*). Human lung NK cells display a differentiated phenotype and also have the capacity to respond to viral infections such as IAV (*16*, *18*, *19*). In COVID-19, emerging studies have reported low peripheral blood NK cell numbers in patients with moderate and severe disease (*4*, *20*–*23*). Two recent reports assessing the single-cell landscape of immune cells in bronchoalveolar lavage (BAL) fluid of COVID-19 patients have suggested that NK cell numbers are increased at this site of infection (*24*, *25*). However, a detailed map of the NK cell landscape in clinical SARS-CoV-2 infection has not yet been established.

Given the important role of NK cells in acute viral infections, their relatively high presence within lung tissue, as well as the links between NK cell activation, T cell responses, and development of immunopathology, we here performed a detailed assessment of NK cells in patients with moderate and severe COVID-19 disease. Using strict inclusion and exclusion criteria, patients with moderate and severe COVID-19 disease were recruited early during their disease, sampled for peripheral blood, and analyzed by 28-color flow cytometry to assess NK cell activation, education, and presence of adaptive NK cells. The results obtained are discussed in relation to the role of NK cell responses in acute SARS-CoV-2 infection and associated COVID-19 disease immunopathogenesis.

## RESULTS

### Study design, clinical cohort, and general NK cell activation in COVID-19

**Fig. 1:**
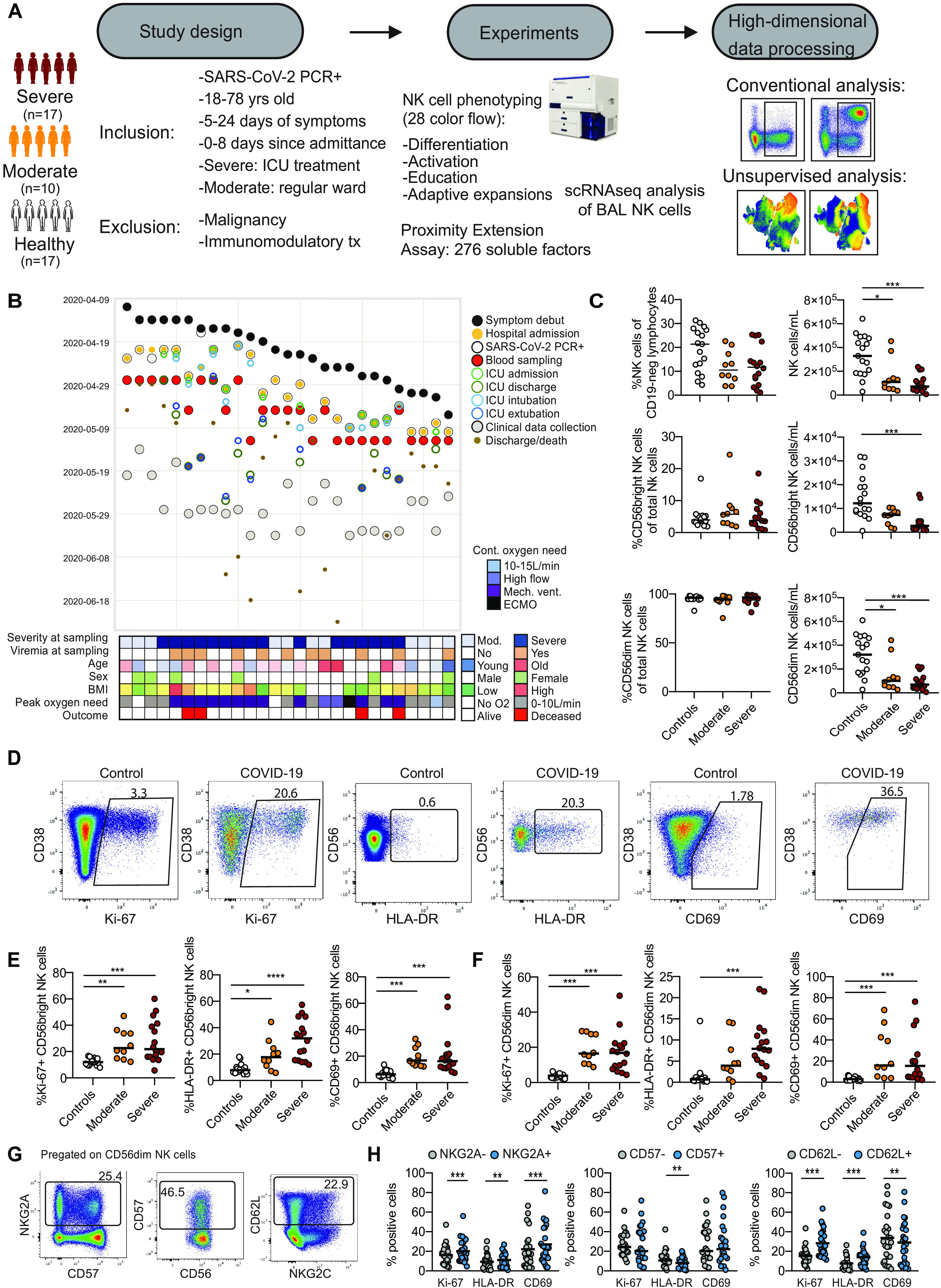
NK cells are robustly activated in moderate and severe COVID-19 disease. (**A**) Schematic overview of study design, inclusion, and exclusion criteria. (**B**) Swimmer plot of symptom debut, hospital admission, and blood sampling in relation to other main clinical events and clinical characteristics. (**C**) Percentages and absolute counts of NK cells and NK cell subsets for healthy controls (*n* = 17), moderate COVID-19 patients (*n* = 10), and severe COVID-19 patients (*n* = 15 to 16). (**D**) Flow cytometry plots of Ki-67, HLA-DR, and CD69 expression on NK cells in one healthy control and one COVID-19 patient. (**E** and **F**) Summary data for expression of the indicated markers in (E) CD56^bright^ and (F) CD56^dim^ NK cells in healthy controls, moderate COVID-19 patients, and severe COVID-19 patients. (**G**) Flow cytometry plots of NKG2A, CD57, and CD62L expression on CD56^dim^ NK cells. (**H**) Summary data for Ki-67, HLA-DR, and CD69 expression within NKG2A^+/−^, CD57^+/−^, or CD62L^+/−^ CD56^dim^ NK cells from all moderate and severe COVID-19 patients (*n* = 24 to 26). In (C) and (E), Kruskal-Wallis test and Dunn’s multiple comparisons test; in (H), Wilcoxon matched-pairs signed rank test. Numbers in flow cytometry plots indicate percentage, and bars represent median (*P < 0.05, **P < 0.01, and ***P < 0.001).

Twenty-seven in-hospital patients with COVID-19 disease (10 moderate and 17 severe) were prospectively recruited after admission to the Karolinska University Hospital as part of a larger immune cell atlas effort (Karolinska COVID-19 Immune Atlas). Strict inclusion and exclusion criteria were used to ensure the setup of homogeneous patient cohorts, including time since symptom debut in relation to hospital admission, and disease severity staging (Fig. 1, A and B). Seventeen healthy controls that were SARS-CoV-2 immunoglobulin G (IgG) seronegative and symptom-free at time of sampling were included as controls. To minimize inter-experimental variability and batch effects between patients and controls, all samples (*n* = 44) were acquired, processed, and analyzed fresh during three consecutive weeks in April and May 2020 at the peak of the COVID-19 pandemic in Stockholm, Sweden (Fig. 1B). More detailed patient characteristics are provided in Materials and Methods and tables S1 and S2.

To study the NK cell response, we analyzed peripheral blood mononuclear cells (PBMCs) with a 28-color NK cell–focused flow cytometry panel (table S3, gating strategy in fig. S1A). No change in total NK cell percentages, or the percentage of CD56^dim^ and CD56^bright^ NK cells, was noted in patients compared with controls. However, the absolute counts of total NK cells, CD56^dim^ NK cells, and CD56^bright^ NK cells were severely reduced in patients compared with controls (Fig. 1C and fig. S1A). NK cell activation was assessed by analyzing expression of Ki-67, HLA-DR (human leukocyte antigen-DR), and CD69 (Fig. 1D). A robust NK cell activation was noted in COVID-19 patients, where the degree of activation was similar in moderate and severe patients (Fig. 1, E and F). CD56^dim^ NK cells undergo continuous differentiation starting from less differentiated NKG2A^+^ and CD62L^+^ cells transitioning toward more terminally differentiated CD57^+^ NK cells (*26*, *27*). A higher percentage of both NKG2A^+^ and CD62L^+^ CD56^dim^ NK cells expressed Ki-67 compared with NKG2A^−^ and CD62L^−^ cells in patients with COVID-19 (Fig. 1, G and H). A subsequent Boolean analysis, simultaneously taking coexpression patterns of NKG2A, CD62L, and CD57 into account, revealed that the NK cell response in patients with COVID-19 occurred primarily among less differentiated CD62L^+^ NK cells (fig. S1B).

Together, using strict inclusion and exclusion criteria and patient recruitment during a defined period of time, we obtained a well-controlled cohort of patients with moderate and severe COVID-19. Peripheral blood NK cells in these patients were robustly activated compared with healthy controls. The general degree of activation was, however, not directly associated with disease severity.

### Phenotypic assessment of NK cells in COVID-19

**Fig. 2:**
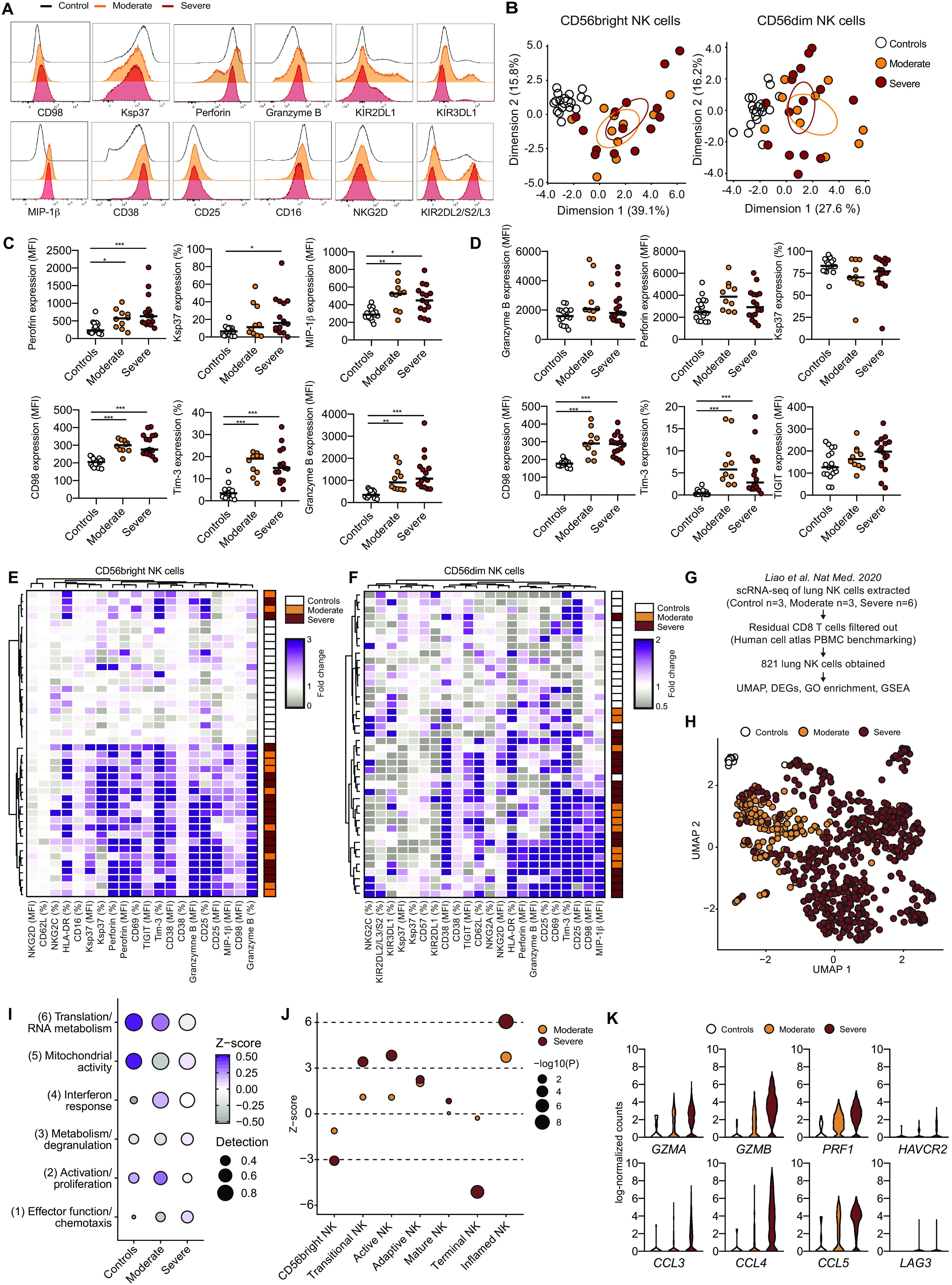
Detailed analysis of NK cell activation in COVID-19 disease. (**A**) Expression of indicated proteins on CD56^dim^ NK cells. (**B**) PCA of the protein expression phenotype of CD56^bright^ and CD56^dim^ NK cells in healthy controls, moderate COVID-19 patients, and severe COVID-19 patients. (**C**) Expression of indicated proteins on CD56^bright^ NK cells in healthy controls (*n* = 17), moderate COVID-19 patients (*n* = 10), and severe COVID-19 patients (*n* = 15). All flow cytometry measurements, including cytokines (MIP-1β and IFN-γ), were performed directly ex vivo without further stimulation. (**D**) Expression of indicated proteins on CD56^dim^ NK cells in healthy controls (*n* = 17), moderate COVID-19 patients (*n* = 10), and severe COVID-19 patients (*n* = 16). (**E** and **F**) Hierarchical clustering heatmaps showing expression of indicated proteins compared with median of healthy controls in (E) CD56^bright^ and (F) CD56^dim^ NK cells. (**G**) Strategy for scRNA-seq analysis of BAL NK cells from controls and COVID-19 patients. (**H**) UMAP of scRNA-seq data for BAL NK cells from the indicated groups. (**I**) Heatmap of indicated gene clusters after gene ontology (GO) enrichment analysis on DEGs. (**J**) *Z* scores of NK cell gene sets after gene set enrichment analysis (GSEA). (**K**) Violin plots of indicated DEGs. In (C) and (D), Kruskal-Wallis test and Dunn’s multiple comparisons test; bars represent median (*P < 0.05, **P < 0.01, and ***P < 0.001).

We next performed a detailed phenotypic assessment of CD56^bright^ and CD56^dim^ NK cells in the COVID-19 patients. By principal components analysis (PCA) of CD56^bright^ (including 26 phenotypic parameters) and CD56^dim^ NK cells (27 phenotypic parameters), patients clustered separately from healthy controls (Fig. 2, A and B). This was, among other phenotypic markers, driven by changes in expression of CD98, Ki-67, and Ksp37 in CD56^bright^ (fig. S2A) and Tim-3, CD98, CD38, CD69, and Ksp37 in CD56^dim^ NK cells (fig. S2B). Flow cytometry analysis by manual gating further revealed increased expression of perforin, Ksp37, MIP-1β (macrophage inflammatory protein-1β), CD98, Tim-3, and granzyme B in CD56^bright^ NK cells from COVID-19 patients, as well as CD98, Tim-3, NKG2C, MIP-1β, and CD62L, among other markers, in CD56^dim^ NK cells (Fig. 2, C and D, and fig. S2, C and D). In line with the activation profile (Fig. 1, E and F), PCA did not highlight an obvious separation between moderate and severe COVID-19 patients (Fig. 2B), and similar results were obtained when unsupervised hierarchical clustering was performed using expression of all studied phenotypic markers within CD56^bright^ and CD56^dim^ NK cells (Fig. 2, E and F). The clustering distinguished healthy controls from COVID-19 patients. Last, the phenotypes of responding (Ki-67 positive) compared with nonresponding (Ki-67 negative) NK cells were compared. This analysis revealed that responding CD56^bright^ NK cells coexpressed higher levels of granzyme B, CD25, HLA-DR, and Ksp37 when compared with nonresponding cells and that responding CD56^dim^ NK cells expressed higher levels of Tim-3 and HLA-DR (fig. S2E).

The activated status of NK cells was also confirmed in BAL from COVID-19 patients by analysis of publicly available single-cell RNA sequencing (scRNA-seq) data (Fig. 2, G and H, and fig. S2F) (*24*). Clustering of differentially expressed genes (DEGs) in moderate and severe patients followed by gene ontology enrichment analysis of DEGs revealed six distinct gene modules where effector functions/chemotaxis, activation/proliferation, and interferon response were enriched in patients compared with controls (Fig. 2I; fig. S2, G and H; and table S4). The interferon response and activation/proliferation signatures were most highly enriched in BAL NK cells from patients with moderate disease, whereas severe patients had a higher effector function gene signature (Fig. 2I and fig. S2, G and H). Gene set enrichment analysis further highlighted NK cells from BAL fluid of severe patients to display an activated and inflamed profile (Fig. 2J and fig. S2I). Last, several of the proteins that were increased in peripheral blood NK cells from COVID-19 patients (Fig. 2, C to F, and fig. S2, C and D) were also identified as DEGs in BAL NK cells of COVID-19 patients as compared with controls, including *GZMB* (granzyme B), *PRF1* (perforin), *HAVCR2* (Tim-3), and *CCL4* (MIP-1β) (Fig. 2K). In summary, peripheral blood CD56^bright^ and CD56^dim^ NK cells displayed an activated effector phenotype that is similar in nature in moderate and severe COVID-19 disease and that could be corroborated by analysis of NK cells from BAL fluid of COVID-19 patients.

### Impact of KIR expression and NK cell education on the NK cell COVID-19 response

CD56^dim^ NK cell function is regulated by inhibitory receptors. Subsets of these cells expressing inhibitory receptors in the presence of self-HLA class I ligands are educated and thus more functional (*28*, *29*). We next evaluated the impact of expression of inhibitory KIRs (killer cell immunoglobulin-like receptors) and NKG2A as well as of NK cell education on the acute COVID-19 response (fig. S3A and table S5). No significant differences were found in the fractions of NK cells expressing different combinations of inhibitory KIRs and NKG2A in COVID-19 patients as compared with controls (fig. S3B). Furthermore, the size of CD56^dim^ NK cell subsets educated via KIRs or NKG2A was also similar in patients and controls (fig. S3C). Last, we assessed the NK cell response by measuring Ki-67 up-regulation in CD56^dim^ NK cell subsets, either solely based on KIR and NKG2A expression or also by integrating information on major histocompatibility complex (MHC) class I ligand haplotypes (fig. S3, D to F). This comparison indicated that the NK cell response in acute COVID-19 occurred independently of inhibitory KIR expression and NK cell education status.

### Increased frequency of adaptive NK cells in severe COVID-19

**Fig. 3:**
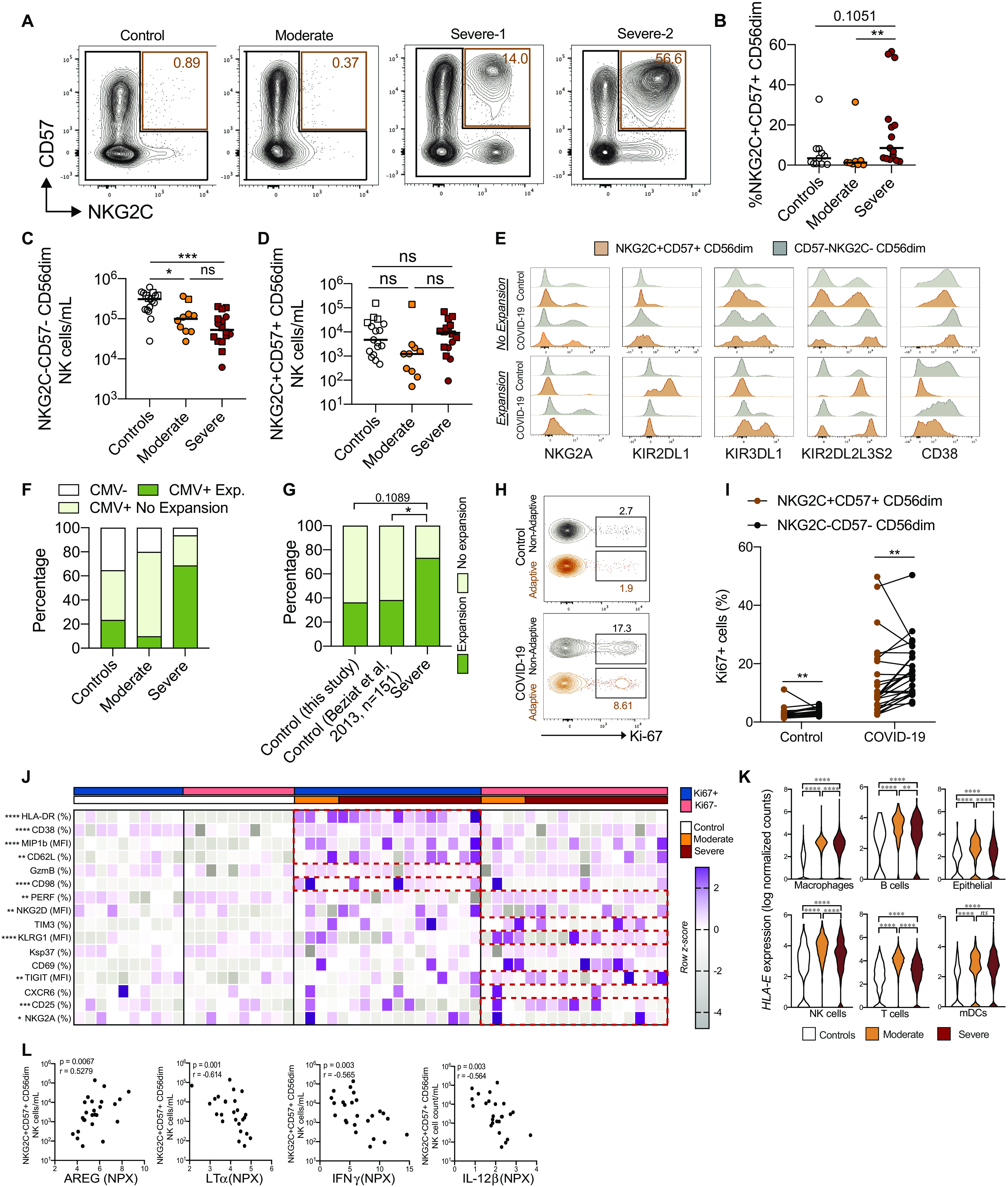
Increase in adaptive NK cells in severe COVID-19 disease. (**A**) CD57 and NKG2C expression on CD56^dim^ NK cells in indicated experimental groups. (**B**) Percentage of NKG2C^+^CD57^+^ cells in CMV-seropositive controls (*n* = 11), moderate COVID-19 patients (*n* = 8), and severe COVID-19 patients (*n* = 15). (**C** and **D**) Absolute counts of the indicated subsets in controls (*n* = 17), moderate COVID-19 patients (*n* = 10), and severe COVID-19 patients (*n* = 16). Squares and circles represent individuals with and without NK cell adaptive expansions, respectively. (**E**) Representative histograms of the indicated protein expression in NKG2C^+^CD57^+^ and NKG2C^−^CD57^−^ CD56^dim^ NK cells. (**F** and **G**) Individuals from the indicated groups having or not having adaptive NK cell expansions. (**H** and **I**) Representative plots and summary data for Ki-67 expression in NKG2C^+^CD57^+^ and NKG2C^−^CD57^−^ CD56^dim^ NK cells in controls (*n* = 17) and COVID-19 patients (*n* = 26). (**J**) Expression *z* score of indicated proteins in NKG2C^+^CD57^+^ NK cells from controls (*n* = 10) and COVID-19 patients (*n* = 17). Red boxes highlight the markers with significantly different expression in the Ki-67^+^ or Ki-67^−^ fraction. (**K**) *HLA-E* expression from scRNA-seq data of the indicated cell types. (**L**) Spearman correlation between NKG2C^+^CD57^+^ CD56^dim^ NK cell numbers and the indicated soluble factors in COVID-19 patients. In (B) to (D), Kruskal-Wallis test followed by Dunn’s multiple comparisons test; in (F) and (G), Fisher’s exact test; in (I) to (J), Wilcoxon matched-pairs signed rank test; in (K), pairwise comparisons (see Materials and Methods). Bars represent median (*P < 0.05, **P < 0.01, ***P < 0.001, and ****P < 0.0001). ns, not significant.

Our analyses so far revealed robust activation of NK cells with an effector phenotype in COVID-19 patients. The response appeared largely independent of disease severity because few differences were observed when comparing moderate and severe patients. However, we noticed that severe patients had increased frequencies of NKG2C^+^ NK cells (fig. S2D). This indicated an increased frequency of adaptive NK cells, which are often associated with cytomegalovirus (CMV) infection and can expand when CMV-seropositive patients undergo other severe acute viral infections (*13*, *30*, *31*). Adaptive NK cells are characterized by a terminally differentiated phenotypic signature, and the vast majority of them coexpresses NKG2C and CD57 (*30*). In severe, but not in moderate COVID-19 patients, we detected higher frequencies of NKG2C^+^CD57^+^ CD56^dim^ NK cells, as compared with healthy controls (Fig. 3, A and B). Moreover, although the absolute number of NKG2C^−^CD57^−^ cells decreased in COVID-19 patients, NKG2C^+^CD57^+^ NK cells remained stable even in patients with severe disease (Fig. 3, C and D). A deeper phenotypic analysis of NKG2C^+^CD57^+^ NK cells, as compared with their NKG2C^−^CD57^−^ counterparts, highlighted the modulation of several maturation and activation-related markers, for instance, NKG2A and CD38 (Fig. 3E and fig. S4A). By applying an arbitrary threshold based on the percentage of NKG2C^+^CD57^+^ NK cells over the CD56^dim^ population (here set to >5%; fig. S4B), we were able to identify patients where NKG2C^+^CD57^+^ NK cells displayed a phenotypic pattern largely overlapping with that observed in adaptive NK cells (Fig. 3E) (*30*, *32*). The expansion of adaptive NK cells was confined to CMV-seropositive individuals (Fig. 3F and table S5). Our strategy to identify expansions in the smaller healthy control group showed good correspondence with a previous study assessing similar expansions in a large cohort of more than 150 healthy individuals (Fig. 3G) (*30*). About two-thirds of CMV-seropositive severe COVID-19 patients had such an expanded adaptive NK cell population compared with one-third in controls and even fewer in the moderate COVID-19 patients (Fig. 3, F and G, and fig. S4C). Thirteen of 16 severe COVID-19 patients were negative for CMV DNA in serum (table S5), indicating that the increased proportion of adaptive NK cells in severe patients did not depend on CMV reactivation secondary to COVID-19. In line with this hypothesis, neither the percentage nor absolute counts or the fraction of proliferating NKG2C^+^CD57^+^ NK cells showed any correlation with CMV IgG levels detected in patient sera (fig. S4D and table S5). The clinical picture was similar in severe patients with and without adaptive NK cell expansions (fig. S4, E and F, and tables S6 and S7).

NKG2C^+^CD57^+^ NK cells found in COVID-19 patients displayed signs of proliferation, although at lower levels compared with NKG2C^−^CD57^−^ NK cells (Fig. 3, H and I, and fig. S4, H to J). Responding NKG2C^+^CD57^+^ NK cells expressed higher levels of HLA-DR, CD38, CD62L, and MIP-1β but showed lower expression of NKG2A, NKG2D, TIGIT, and CD25 as compared with nonresponding NKG2C^+^CD57^+^ NK cells (Fig. 3J and fig. S4K). Next, we set out to more specifically address what was driving the emergence of adaptive NK cell expansions. Reanalysis of published scRNA-seq data revealed that both immune and nonimmune cell types displayed up-regulated expression of genes encoding HLA-E and HLA-A, HLA-B, and HLA-C in BAL fluid of moderate and severe COVID-19 patients as compared with controls (Fig. 3K and fig. S4L). The number of NKG2C^+^CD57^+^ NK cells was inversely correlated with several soluble factors, including lymphotoxin-alpha (LT-α), IL-12β, interferon-γ (IFN-γ), and amphiregulin (Fig. 3L). However, when specifically comparing soluble factors in severe patients with and without expansions, only a limited number of factors were differentially expressed (fig. S4M). In line with this, associations between phenotypic features and soluble factors were, in most cases, similar for adaptive and nonadaptive NK cells (fig. S4N). Together, severe, but not moderate, COVID-19 disease is associated with higher frequencies of adaptive NK cells that display signs of proliferation and activation without detectable concurrent CMV reactivation.

### Dimensionality reduction analysis of NK cells separates severe from moderate patients

**Fig. 4:**
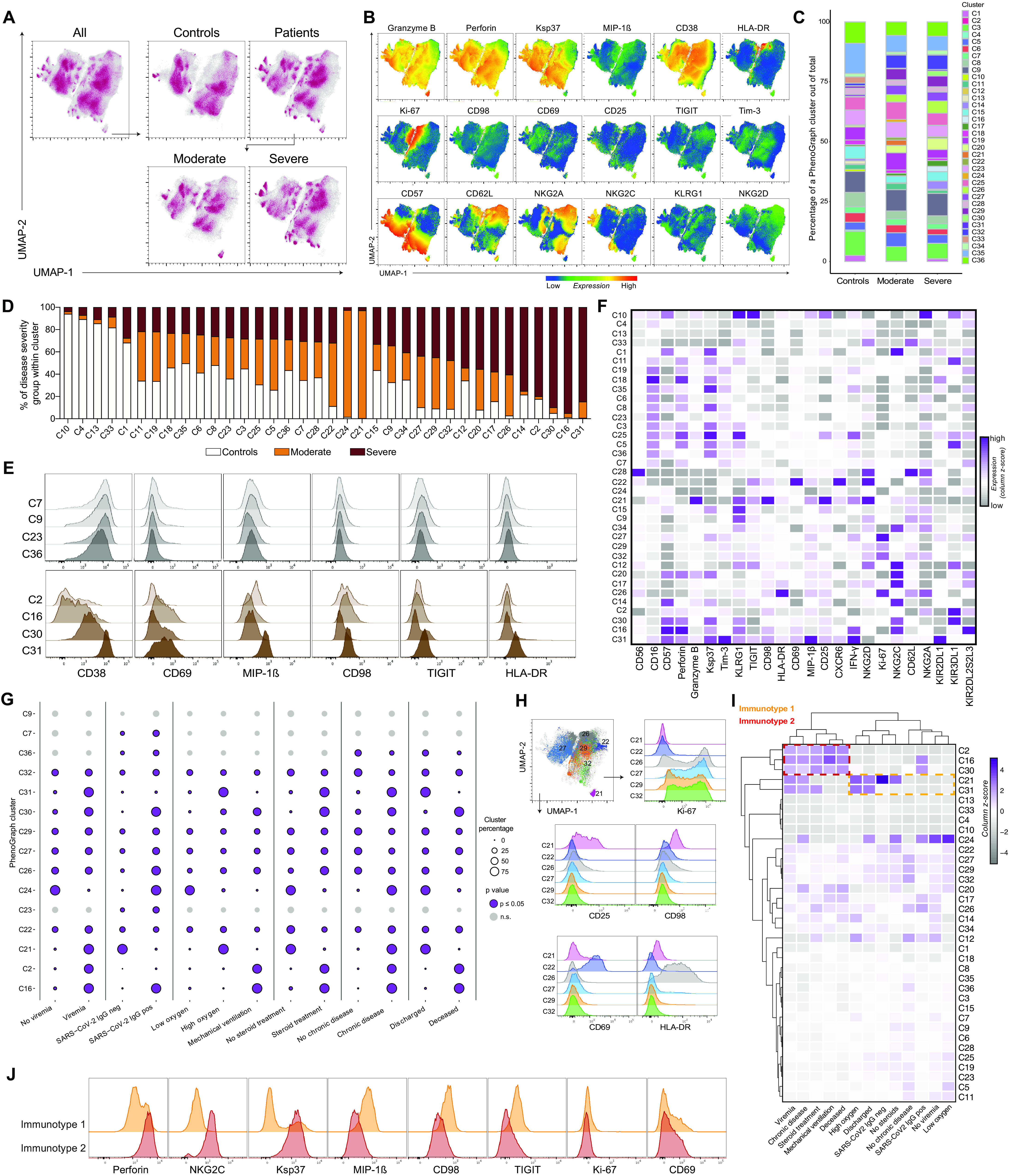
Automated analysis of NK cells in COVID-19 identifies putative NK immunotypes differentially enriched in two main groups of patients. (**A**) UMAP of all events, of controls and patients, and of patients split into moderate and severe. (**B**) Representative expression of phenotypic markers on all cells in the UMAP. (**C**) Percentage of 36 PhenoGraph clusters within total cells of indicated groups. (**D**) Relative abundance of control, moderate, and severe groups within each PhenoGraph cluster. (**E**) Expression of selected markers from representative PhenoGraph clusters that did not display significant differential relative abundance between healthy, moderate, and severe groups (top) and significant clusters that were most highly abundant in severe COVID-19 patients (bottom). (**F**) Expression of phenotypic markers across PhenoGraph clusters (as column *z* score of median expression values). (**G**) Percentage of NK cell clusters from COVID-19 patients stratified according to the indicated clinical categorical parameters. Purple circles with a border indicate significant PhenoGraph clusters in a particular comparison (*P* ≤ 0.05), and light gray circles without a border indicate nonsignificant clusters (Materials and Methods and table S8). Vertical lines indicate separate comparisons. (**H**) Selected PhenoGraph clusters overlaid on the UMAP and representative flow cytometry histograms showing expression of the indicated markers within the clusters. (**I**) Hierarchical clustering of PhenoGraph clusters and clinical categorical parameters. The heatmap was calculated as column *z* score of cluster percentages. Putative NK immunotypes are indicated. (**J**) Expression of separating and nonseparating markers within the NK cell immunotype 1 and 2 clusters.

Except for adaptive NK cell expansions being more prevalent in severe COVID-19 patients, manual gating–based flow cytometry analysis did not identify features specific to either moderate or severe COVID-19 patients. In an additional attempt to identify such features through an unsupervised approach, we performed uniform manifold approximation and projection (UMAP) analysis on all patients and controls (Fig. 4, A and B, and fig. S5). This revealed distinct topological regions between patients and controls (Fig. 4A). We applied PhenoGraph to our samples to further describe NK cell subpopulations and to quantify differences in their relative abundance between COVID-19 patient groups. PhenoGraph clustering identified 36 distinct clusters (Fig. 4C). About half of these clusters were equally shared between controls and moderate and severe patients (Fig. 4D), and some clusters were patient specific. Furthermore, this analysis also revealed clusters that contained NK cells predominantly from controls, moderate COVID-19 patients, or severe COVID-19 patients (Fig. 4, C and D). The phenotype of NK cells in clusters with similar relative abundance across the three groups was homogenous, but greater differences were observed when assessing clusters uniquely and significantly enriched in moderate or severe patients (Fig. 4, E and F, and fig. S5D).

Next, each PhenoGraph cluster was stratified within patients on the basis of six clinical categorical parameters (viremia, seroconversion, oxygen need, steroid treatment, underlying comorbidities, and outcome) (Fig. 4G) in the same manner as moderate and severe patient categories were defined (Materials and Methods). Here, most clusters showed a significantly different relative abundance between patient categories and healthy controls in at least one of the clinical parameter–defined patient groups (Fig. 4G, fig. S6A, and table S8). For some clusters, the relative abundances were different depending on the clinical parameter. This approach was critical for our understanding, because it enabled us to step away from looking at disease severity on the whole and to instead look into the defining clinical parameters separately. For example, cluster 21 with high CD25 and CD98 expression (Fig. 4H) was almost completely absent from patients who were on mechanical ventilation and the ones who died from COVID-19. Conversely, CD69-expressing cluster 22 and the four most highly Ki-67–expressing clusters accounted for a similar percentage across all patient categories in our dataset (Fig. 4H and fig. S6, B to F). Hierarchical clustering of PhenoGraph clusters’ relative abundance across clinical categorical parameters revealed the presence of two “immunotypes” in patients. One of these contained categorical parameters attributed to milder disease course, and the other one was enriched for viremia, chronic underlying diseases, steroid treatment, mechanical ventilation, and fatal outcome (Fig. 4I). A similar clustering was obtained when moderate and severe disease status was included (fig. S6G). Last, the NK cell phenotypes associated with moderate (immunotype 1) versus severe (immunotype 2) disease were assessed. This revealed up-regulated MIP-1β, CD98, and TIGIT in the moderate immunotype and conversely high expression of perforin, NKG2C, and Ksp37 in the severe immunotype, whereas the activation markers Ki-67 and CD69 remained unchanged (Fig. 4J). High perforin, NKG2C, and Ksp37 are features of adaptive NK cell expansions that were also associated with severe disease (Fig. 3). In summary, unsupervised high-dimensional analysis of the NK cell response in COVID-19 disentangles NK cell phenotypes associated with disease severity.

### CD56^bright^ NK cell arming is associated with disease severity

**Fig. 5:**
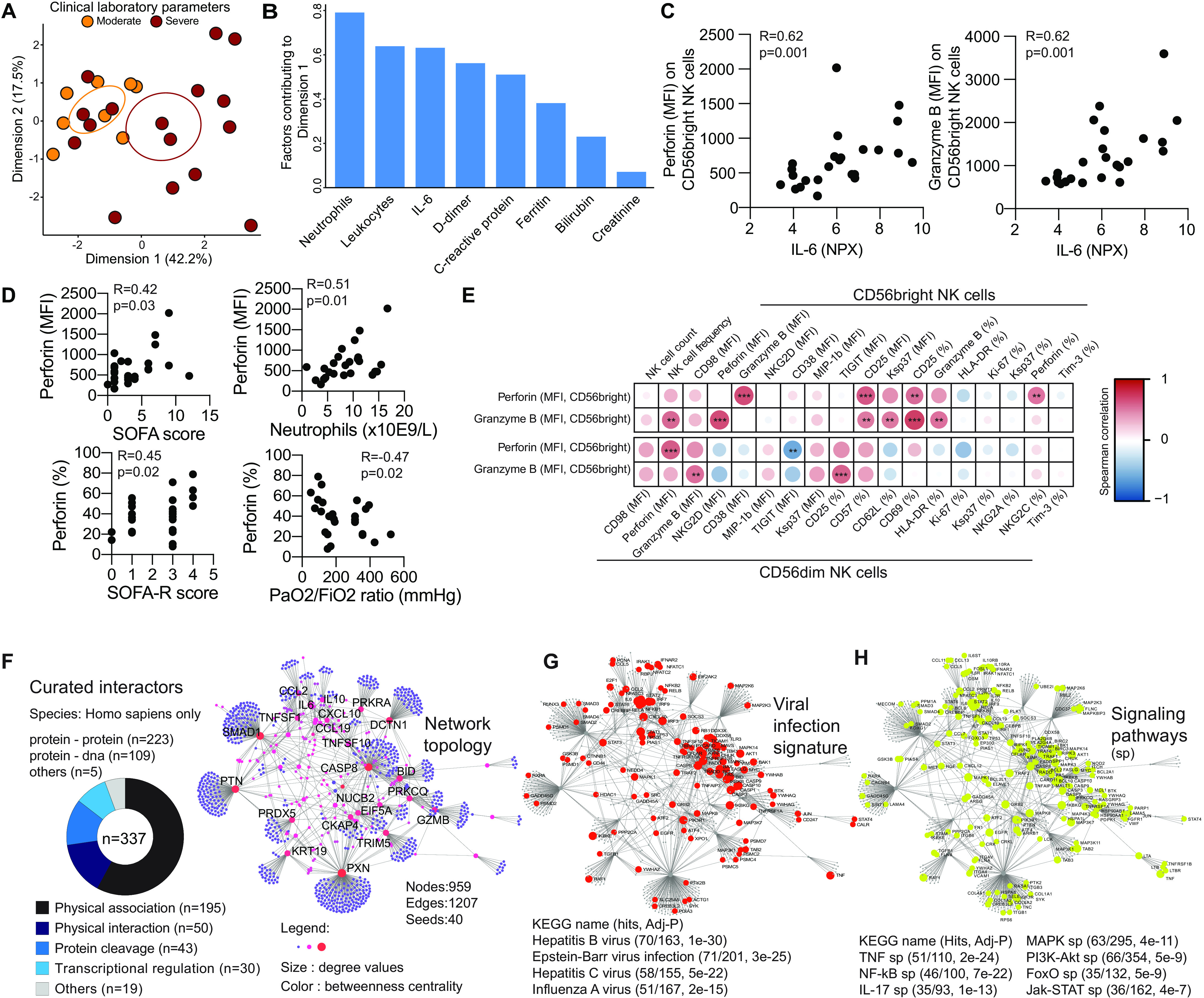
Arming of CD56^bright^ NK cells associate with COVID-19 disease severity. (**A**) PCA of COVID-19 patients based on clinical laboratory parameters. (**B**) Bar plot showing the clinical laboratory parameters contributing to dimension 1 (dim 1) in (A). (**C**) Spearman correlations between the indicated CD56^bright^ NK cell phenotypic parameters in COVID-19 patients and serum IL-6 levels (*n* = 24). (**D**) Spearman correlations between the indicated CD56^bright^ NK cell phenotypic parameters and clinical parameters (*n* = 25). (**E**) Correlation matrix showing Spearman correlations between perforin and granzyme expression (MFI) on CD56^bright^ NK cells and the indicated other NK cell phenotypic parameters (*n* = 25). Color indicates *R* value, and asterisks indicate *P* values. (**F**) Topology and content of the protein-protein interaction network driven by soluble factors (seeds) correlated with perforin and granzyme B expression in CD56^bright^ NK cells. Superimposition of nodes involved in (**G**) viral infection and (**H**) signaling pathways on the identified protein-protein interaction network.

Last, we performed a more detailed analysis of the NK cell response in relation to clinical laboratory parameters and clinical scoring systems. PCA of moderate and severe patients based on clinical laboratory parameters revealed separation between the two groups (Fig. 5A). This was primarily driven by high systemic levels of neutrophils, IL-6, D-dimer, and C-reactive protein, clinical laboratory data typically associated with severe disease (Fig. 5B). Out of the altered NK cell phenotypic parameters (Fig. 2 and fig. S2), the expression levels of perforin and granzyme B in CD56^bright^ NK cells correlated with IL-6 levels in the patients (Fig. 5C). Perforin in CD56^bright^ NK cells in the COVID-19 patients also positively correlated with sequential organ failure assessment (SOFA) and SOFA-R scores as well as neutrophil count and negatively with PaO_2_/FiO_2_ ratio (Fig. 5D). Similarly, patients with ongoing SARS-CoV-2 viremia in serum also presented with higher CD56^bright^ NK cell perforin expression, and these viremic patients had more severe disease (fig. S7A). The up-regulation of perforin and granzyme B on CD56^bright^ NK cells (Fig. 2) was further positively associated with a general activation and up-regulation of effector molecules within CD56^bright^ and CD56^dim^ NK cells and inversely correlated with expression of the inhibitory checkpoint molecule TIGIT (Fig. 5E and fig. S7, B and C). Hence, the association with high perforin was in line with the severe immunotype identified through unsupervised high-dimensional analysis (Fig. 4). Thus, integration of clinical laboratory parameters into phenotypical NK cell analysis revealed that arming of CD56^bright^ cells with cytotoxic molecules correlated with parameters associated with a severe disease course.

### Protein-protein interaction network driving CD56^bright^ NK cell arming in COVID-19

To better understand the significance of the activated CD56^bright^ NK cell phenotype and its association with disease severity, we took advantage of an extensive characterization of soluble serum proteins that had been performed on the same 27 patients through the Karolinska COVID-19 Immune Atlas effort. Of the 276 quantified soluble factors, the concentrations of 58 were significantly associated with perforin and granzyme B expression on CD56^bright^ cells and 9 showed strong, primarily positive, correlations (fig. S7D). To gain insight into the biological basis of the peripheral protein signature that correlated with CD56^bright^ NK cell activation, we analyzed protein-protein interaction networks. We identified 337 interactions (table S9) that were integrated into a network of 959 elements (nodes; Fig. 5F and table S10). Many of these related to viral infections, suggesting redundancy between different viral infections in the NK cell activation mode (Fig. 5G and table S11). In addition, several distinct signaling pathways potentially involved in driving the CD56^bright^ NK cell response in COVID-19 together with the soluble factors were identified (Fig. 5H, fig. S7F, and table S11). This included nuclear factor κB (NF-κB), phosphatidylinositol 3-kinase (PI3K)–Akt, and FoxO signaling (Fig. 5H). Together, our findings indicate that NK activation pathways could be shared with other viral infections, connecting NK cell–specific phenotypic traits to components of the systemic inflammatory milieu related to COVID-19 disease pathogenesis.

## DISCUSSION

Using high-dimensional flow cytometry, integration of soluble factor analysis, and complementing with the analysis of publicly available scRNA-seq data, we here characterized the host NK cell response toward SARS-CoV-2 infection in moderate and severe COVID-19 patients in the circulation. Results revealed low NK cell numbers but a robustly activated NK cell phenotype in COVID-19 patients. Peripheral blood NK cell activation states were mirrored in transcriptional signatures of BAL NK cells of COVID-19 patients from another cohort (*24*). Unsupervised high-dimensional analysis furthermore revealed clusters of NK cells that were linked to the disease status. Last, severe hyperinflammation, defined by clinical parameters, was associated with a high presence of adaptive NK cell expansions and arming of CD56^bright^ NK cells. These systemic immune changes were linked to a soluble factor protein interaction network displaying a canonical viral activation signature and distinct signaling pathways. Together, the results provide a comprehensive map of the landscape of NK cell responses in SARS-CoV-2–infected COVID-19 patients at early stages of disease and yield insights into host responses toward viral infections and associated disease pathology.

NK cells are known to rapidly respond during diverse acute viral infections in humans including those caused by dengue virus, hantavirus, tick-borne encephalitis virus, and yellow fever virus (*13*–*15*, *33*). Although a similarly detailed analysis of NK cells has not been performed in acute SARS-CoV-2 infection causing COVID-19, early reports from the pandemic have indicated low circulating NK cell numbers in patients with moderate and severe disease (*20*, *21*, *23*). Those reports are in line with what is reported here. Transiently reduced NK cell numbers during the acute phase of infections have also been shown in SARS-CoV-1 infection (*34*) and in acute hantavirus infection (*13*). The magnitude of the present detected NK cell response, with a quarter of the circulating cells either displaying signs of proliferation or activation, mirrors the responses observed during acute dengue fever and in acute hantavirus infection (*13*, *14*). The present in-depth phenotypic assessment of NK cells in moderate and severe COVID-19 patients revealed an activated phenotype with up-regulated levels of effector molecules and chemokines, activating receptors, and nutrient receptors. We also observed signs of inhibitory immune checkpoint receptor up-regulation through increased levels of TIGIT and Tim-3. Hence, the present results confirm and extend, at protein level, what was recently reported for peripheral blood NK cells using scRNA-seq (*20*).

Human NK cells are enriched in the lung compared with peripheral blood (*18*, *19*). COVID-19 patients display significant immune activation in the respiratory tract, where moderate disease is associated with a high number of T cells and NK cells, whereas neutrophils and inflammatory monocytes are enriched at these sites in patients with severe disease (*24*, *25*, *35*). By analyzing publicly available scRNA-seq data on BAL NK cells from COVID-19 patients (*24*), we found these cells to be strongly activated. The interferon response appeared stronger in BAL NK cells from moderate COVID-19 patients. This is in line with severe COVID-19 associating with a blunted interferon response (*6*). We further confirmed that a similarly activated phenotype of NK cells was present in BAL fluid, as was observed in peripheral blood, including high expression of effector molecules and chemokines. NK cell homing to the site of infection is important for pathogen clearance in murine models, where several chemokine receptors have been shown to mediate NK cell tissue homing (*36*–*39*). The reduced numbers of NK cells that we observed in circulation might, to some extent, reflect a redistribution to the lung. Chemotaxis was another gene ontology module that, together with cytotoxicity, was enriched in BAL NK cells of severe COVID-19 patients. In addition, BAL fluid from COVID-19 patients contains elevated levels of chemokines that potentially could attract NK cells, including CCL3, CCL3L1, CCL4, CXCL9, CXCL10, and CXCL11 (*24*). Future work should dissect NK cell trafficking to the lung in COVID-19 in more detail and in relation to COVID-19 disease severity. To this end, indirect and related findings suggest that NK cells may have a role in the early host immune response toward SARS-CoV-2 at the site of infection.

This is the first time an increase in adaptive NK cells is described in COVID-19 patients. This feature was almost exclusively found in SARS-CoV-2–infected patients with severe disease. The percentage of adaptive NK cell expansions in the present healthy control cohort was comparable to previous findings on larger healthy CMV-seropositive human cohorts (*30*, *40*), strengthening our observations in COVID-19 patients. The lack of any correlation between the emergence of adaptive NK cell expansions and CMV IgG titers as well as the occurrence of CMV reactivation due to possible COVID-19–induced immune dysfunction is noteworthy, as adaptive NK cell expansions have been shown to associate with CMV antiviral immunity (*32*, *40*). The absence of an ongoing CMV-directed immune response is further corroborated by the lack of proliferation observed in CMV-specific CD8^+^ T cells in acute COVID-19 patients (*8*). These findings might suggest a direct and virus-specific involvement of adaptive NK cells during SARS-CoV-2 infection, although future studies will be required to formally prove such hypotheses. Furthermore, future work should, in more detail, assess the phenotype and function of adaptive NK cells in COVID-19 compared with those found only in CMV-seropositive individuals, as well as the possible long-term maintenance of adaptive cells after recovery from SARS-CoV-2 infection.

Whereas a recent study reported an increase in NKG2C expression and a corresponding decreased expression of the adaptive NK cell–related marker FcεR1ɣ (*41*), we here provide a deep dissection of the adaptive NK cell phenotype, highlighting that despite their lower responsiveness to cytokines (*40*), a significant percentage of adaptive NK cells (up to 45%) actively proliferate in COVID-19 patients, albeit at a lower rate compared with nonadaptive NK cells. Moreover, in stark contrast to the robust numeric reduction observed of nonadaptive NK cells and in many other lymphocyte subsets during acute SARS-CoV-2 infection (*42*, *43*), adaptive NK cell numbers were unchanged in peripheral blood in severe COVID-19 patients. This suggests that their relative accumulation in blood in the course of the SARS-CoV-2 infection might be related to their higher resistance to cytokine-induced apoptosis (*40*) or to a different sensitivity to chemotactic signals compared with nonadaptive NK cells. More mature CD62L^−^NKG2A^−^ CD56^dim^ NK cells express higher levels of CX3CR1 compared with less mature NKG2A^+^ and CD62L^+^ CD56^dim^ NK cells (*44*), and the CX3CR1 ligand CX3CL1 was one of the few chemokines that were not induced in BAL from COVID-19 patients (*25*). Conversely, CXCR3, ligands for which were up-regulated in BAL of COVID-19 patients (*24*), is expressed at low levels by mature CD57^+^ CD56^dim^ NK cells (*45*). As adaptive CD56^dim^ NK cells display a highly mature phenotype, it is likely that they express high CX3CR1 but low CXCR3 compared with less mature nonadaptive CD56^dim^ NK cells and might thus be more likely to maintain in circulation.

Adaptive NK cells can sense target cells via NKG2C directly recognizing HLA-E. Here, we found up-regulated *HLA-E* in immune and stromal cells in BAL fluid of COVID-19 patients, suggesting a receptor-ligand driven expansion of adaptive NK cells. Expansion of adaptive NK cells in CMV-seropositive patients was previously reported in acute hantavirus infection (*13*) and was associated with the up-regulation of both class I MHC and HLA-E on the virus-infected cells. The absence of strong links between soluble serum proteins and adaptive NK cell expansions suggests that their phenotype is instead driven by receptor-ligand interactions in COVID-19. In CMV infection, virus-encoded peptides presented on HLA-E serve as a key activator of NKG2C^+^ cells and, together with cytokines, contribute to their expansion and differentiation (*46*). The potential relevance of the NKG2C–HLA-E axis in the context of SARS-CoV-2 infection is highlighted by a recent study showing an increased prevalence of the allelic variant NKG2C^del^, associated with a reduced NKG2C expression, among severe COVID-19 patients, compared with the healthy population (*47*). Moreover, a higher frequency of the HLA-E*0103 allele was found in intensive care unit (ICU)–hospitalized patients from the same cohort. Together, our working hypothesis is that a higher percentage of NKG2C^+^ NK cells could be functionally required to unleash NK cell antiviral activity in SARS-CoV-2 infection, particularly in more severe patients, whereas genetic predisposition leading to the reduction of NKG2C could cause more severe clinical manifestations.

We included viremia and other clinical categorical data that associate with disease severity in the UMAP analysis of NK cells in COVID-19 patients. These integrated immune signatures allowed us to, without a priori knowledge of the disease stage, identify two immunotypes associated with COVID-19 severity. The immunotype that was linked to severe disease displayed high expression of perforin, Ksp37, and NKG2C. This corroborated our finding of increased adaptive NK cells in severe COVID-19 because a link between NKG2C and disease severity was discovered in two independent types of analyses. Similarly, both perforin and Ksp37 were part of the compound phenotype of armed CD56^bright^ NK cells that we found associated with severe disease, and the scRNA-seq analysis identified effector function as the gene ontology module most highly detected in severe patients. The exact role of Ksp37 in the (adaptive) NK cell response to COVID-19 needs to be determined in future studies. The immunotype linked to moderate disease, on the other hand, was associated with higher expression of MIP-1β, CD98, and TIGIT. The association between high expression of the inhibitory checkpoint TIGIT and moderate disease was strengthened by the fact that the armed CD56^bright^ NK cell phenotype negatively correlated with TIGIT expression. Future work should address whether containment of NK cells by inhibitory checkpoints could aid in containing hyperinflammation and severe COVID-19 disease.

A strong association between arming of NK cells and disease severity was observed, which was coupled not only to IL-6 levels but also to several clinical disease severity scores. Our protein-protein network linked to this arming revealed that interactions active in COVID-19 were shared with other viral infections such as hepatitis B and IAV. Along the same lines, arming of CD56^bright^ NK cells primarily occurred in COVID-19 patients with ongoing SARS-CoV-2 viremia, suggesting this to be a common host response feature of NK cells against several viral infections. Furthermore, NF-κB signaling was strongly enriched in the protein-protein network. NF-κB signaling is important for transcription of the genes encoding perforin and granzyme B in NK cells (*48*, *49*), thus possibly providing a pool of mRNAs that equip CD56^bright^ NK cells with cytotoxicity potential (*50*) and final signals for cytotoxicity upon target cell recognition (*51*). Last, IL-6 associated with PI3K-Akt and FoxO signaling in NK cells. Both of these pathways are known to coordinate the cell cycle and have previously been reported to be activated in NK cells during acute dengue infection in humans (*14*). Together, our protein-protein network analysis of soluble factors provided insights into shared features between activated NK cells in diverse viral infections and signaling pathways contributing to the arming of CD56^bright^ NK cells.

Although certain aspects of the NK cell response, including emergence of adaptive NK cell expansions and arming of CD56^bright^ NK cells, appeared specific to severe COVID-19, the contribution of these features to the disease pathogenesis needs to be confirmed in relation to the high levels of proinflammatory cytokines present in these patients (*52*). Thus, future studies should aim to longitudinally assess the NK cell response from very early in the acute phase of infection and simultaneously map other innate immune cells, such as monocytes and neutrophils, that are dysregulated in severe COVID-19 (*52*, *53*). Overall, we observed a robust NK cell response toward SARS-CoV-2 infection and specific features unique to severe COVID-19 and hyperinflammation, providing a base for understanding the role of NK cells in patients with COVID-19.

## MATERIALS AND METHODS

### Patient characteristics

SARS-CoV-2 RNA^+^ patients with moderate or severe COVID-19 disease admitted to the Karolinska University Hospital, Stockholm, Sweden, were recruited to the study. COVID-19 patients were sampled 5 to 24 days after symptom debut and 0 to 8 days after being admitted to the hospital. Patients classified as having moderate COVID-19 disease had oxygen saturation of 90 to 94% or were receiving 0.5 to 3 liters/min of oxygen at inclusion. Patients with severe COVID-19 disease were treated in an ICU or a high dependency unit, requiring either noninvasive or mechanical ventilation. Included patients were between 18 and 78 years old. For both groups, patients with current malignant disease and/or ongoing immunomodulatory treatment before hospitalization were excluded. The patients were further described by the SOFA score (*54*) and the National Institutes of Health (NIH) ordinal scale (*55*). Healthy controls were SARS-CoV-2 IgG seronegative at time of inclusion, median age was 50 to 59 years, and 11 of 17 were male (65%). All clinical laboratory analyses, including serology and polymerase chain reaction (PCR) for CMV and SARS-CoV-2, were performed using clinical routine assays in the clinical laboratories at the Karolinska University Hospital, Stockholm, Sweden. The study was approved by the Swedish Ethical Review Authority, and all patients gave informed consent. For detailed clinical information, see tables S1 and S2.

### Cell preparation and flow cytometry

Venous blood samples were collected in heparin tubes and PBMCs isolated using Ficoll gradient centrifugation. PBMCs were thereafter stained fresh with the antibody mix (for antibodies, see table S3). Live/Dead cell discrimination was performed using fixable viability dye (Invitrogen). Cells were permeabilized with the Foxp3/Transcription Factor Staining Kit (eBioscience). After staining, cells were fixed with 1% paraformaldehyde for 2 hours before being acquired on a BD FACSymphony with 355-, 405-, 488-, 561-, and 640-nm lasers. In addition, 50 μl of whole blood from each patient and healthy control was used with BD Trucount Tubes to obtain absolute counts of NK cells in the blood according to the instructions from the manufacturer.

### Flow cytometry data analysis

FCS3.0 files were exported from the FACSDiva and imported into FlowJo v.10.6.2 for subsequent analysis. The following plug-ins were used: FlowAI (2.1), DownSample (3.2), UMAP (3.1), and PhenoGraph (2.4). First, the data were preprocessed using FlowAI (all checks, second fraction FR = 0.1, alpha FR = 0.01, maximum changepoints = 3, changepoint penalty = 500, and dynamic range check side = both) to remove any anomalies present in the FCS files. Then, the compensation matrix for the 28-color flow cytometry panel was generated using AutoSpill (*56*) and applied to files. Dataset as such was used for the downstream analysis in both manual gating and automated analysis. For the automated analysis, events were first downsampled from the NK gate across all samples using DownSample (fig. S5, A and B). Clinical parameter categorical values for each sample were added to downsampled populations as metadata to enable identification of these groups, and these were then concatenated for analysis. UMAP was run using all parameters from the panel except BV510 (Live/Dead, CD14, CD15, and CD19) and phycoerythrin (PE)–Cy5 (CD3). PhenoGraph was run using the same parameters from the panel as UMAP (and *k* = 30). Fifteen thousand cells per sample were exported from the NK gate, apart from six patient samples with fewer events where all cells were taken. When assigning categorical groups formed by different clinical parameters, there was an uneven number of patients represented in each group (e.g., 17 “healthy controls,” 12 “viremic,” and 12 “nonviremic” patients). Over- and underrepresented input groups will be similarly weighted in the PhenoGraph output clusters. Therefore, we normalized the PhenoGraph output clusters to account for the total number of cells from each input group. Certain figures were generated in R (versions 3.6.0 and 3.6.1) with packages factoextra (v1.0.5), RColorBrewer (v1.1-2), ggplot2 (v3.2.1 and v3.3.0), tidyr (v.1.0.2), randomcoloR (v.1.1.0.1), reshape2 (v.1.4.3), viridis (v.0.5.1), and pheatmap (v.10.12).

### KIR-ligand genotyping

The DNeasy Blood & Tissue Kit (QIAGEN) was used to extract genomic DNA from control and patient whole blood (collected in the EDTA blood tubes). DNA was extracted from 100 μl of whole blood according to the manufacturer’s instructions. DNA was precipitated from the eluate obtained after the last step of the DNeasy Blood & Tissue Kit protocol, with 2.5× volume 100% ethanol and 0.1× volume 3 M sodium acetate per reaction, and washed with 70% ethanol. DNA concentration was determined with Qubit 4. KIR-ligand genotyping was performed using the KIR HLA Ligand Kit (Olerup-SSP/CareDx) as per the manufacturer’s instructions.

### Serum protein quantification using proximity extension assay

Sera from all patients were evaluated for soluble factors using proximity extension assay technology (Olink AB, Uppsala), where 276 selected soluble factors were analyzed.

### Strategy to identify adaptive NK cell expansions

CMV-seropositive healthy controls and COVID-19 patients displaying more than 5% of NKG2C^+^CD57^+^ cells within their CD56^dim^ NK cell population were considered to have adaptive NK cell expansions (fig. S5A). In all individuals with adaptive expansions, adaptive NK cells displayed higher (>20%) frequencies of either CD57, CD38, or single KIRs compared with the nonadaptive NK cells (and also in one case high NKG2A). One patient displayed a percentage of adaptive NK cells lower than 5% (3.64%) but was included in the expansion group because coexpression of the other phenotypical markers (differential NKG2A, CD57, CD38, and KIR expression) was in line with what has been described for adaptive NK cells.

### scRNA-seq data analysis

Preprocessed scRNA-seq data were obtained from a published dataset (*24*). Data in h5 format were read using Seurat (v3.1.5), then filtered for zero-variance genes, and size-factor–normalized using scater v1.12.2/scran v1.12.1. CD8 T cells were excluded from downstream NK cell analyses based on *k*-means clustering of CD8 and NK cell markers from the human cell atlas PBMC benchmarking dataset (*57*), followed by renormalization of the data. The top 1000 highly variable genes were identified using scran [ranked by biological variance and FDR (false discovery rate)] and used for UMAP dimensionality reduction. Pairwise differential expression of genes detected in at least 20% of cells was performed using MAST (v1.10.0), and genes with an FDR < 10^−3^ in any comparison were clustered into six clusters (determined by gap statistic) by *k*-means clustering of *z* scores. Gene ontology enrichment of gene clusters was performed using PANTHER overrepresentation tests (release 20200407) using gene ontology database 2020-03-23. Gene set enrichment for bone marrow and blood NK cell signature gene sets (*58*) was performed using MAST against a bootstrapped (*n* = 100) control model of the data, and test statistics were combined using the Stouffer method. Data were visualized using ComplexHeatmap (v2.0.0) and ggplot2 (v3.2.1).

### Network analysis

For network analyses, literature-curated interactions from International Molecular Exchange (IMEx) Consortium interactome database, Innate DB, were used. Generic protein-protein interaction network analysis was performed using Steiner forest network and visualized using NetworkAnalyst3.0.

### Statistical analysis

Statistical significance was determined using GraphPad Prism v8. For comparisons between three groups, one-way analysis of variance (ANOVA) and Kruskal-Wallis test followed by Dunn’s multiple comparisons test were used. For two groups, either parametric or nonparametric matched or nonmatched tests were performed. Where indicated, *z* score of either median fluorescence intensity (MFI) or percentage of marker expression was calculated as follows: $z=\frac{\left(x-\mu \right)}{\sigma }$, where *x* is the raw score, μ is the mean of sample distribution, and σ is the SD. For categorical comparisons, Fisher’s exact test was used. Significant PhenoGraph clusters (*P* ≤ 0.05) were determined by chi-square goodness-of-fit tests comparing the relative abundance of each categorical group in each individual PhenoGraph cluster relative to input. For flow cytometry analysis, expression data for multiple markers (both MFI and percentage of protein-expressing cells) derived from gates containing less than 100 events were excluded from analysis. When analyzing the percentage of Ki-67–expressing cells, data derived from gates containing less than 50 cells were excluded from analysis. More details on the exact statistical tests used are mentioned in the respective text/figure legends.

## SUPPLEMENTARY MATERIALS

immunology.sciencemag.org/cgi/content/full/5/50/eabd6832/DC1 Fig. S1. NK cell differentiation in COVID-19 disease. Fig. S2. NK cell activation in COVID-19 disease. Fig. S3. KIRs and NK cell education in COVID-19 disease. Fig. S4. Adaptive NK cell expansions in COVID-19. Fig. S5. Strategy for UMAP analysis and representative marker expression. Fig. S6. Selected PhenoGraph clusters and their markers are expressed differentially across clinical parameter–defined patient groups. Fig. S7. Correlations between CD56^bright^ NK cell arming, NK cell phenotype, and soluble factors in COVID-19. Table S1. Clinical characteristics of COVID-19 patients. Table S2. Clinical laboratory results of COVID-19 patients. Table S3. Flow cytometry panel. Table S4. Gene ontology analysis of DEGs from scRNA-seq analysis of BAL NK cells in COVID-19 (Excel spreadsheet). Table S5. KIR-ligand and CMV characteristics. Table S6. Clinical laboratory results of all patients with and without adaptive NK cell expansions. Table S7. Clinical laboratory results of severe patients with and without adaptive NK cell expansions. Table S8. Analysis of the observed distribution of PhenoGraph clusters across clinical parameter–defined groups. Table S9. Literature-curated interactions from IMEx Consortium interactome database (Excel spreadsheet). Table S10. Nodes and related degrees and betweenness (Excel spreadsheet). Table S11. Overview of the KEGG pathways (Excel spreadsheet). Table S12. Raw data file (Excel spreadsheet).

## Appendix

View/request a protocol for this paper from *Bio-protocol*.
